# Characterization of MHz pulse repetition rate femtosecond laser-irradiated gold-coated silicon surfaces

**DOI:** 10.1186/1556-276X-6-78

**Published:** 2011-01-12

**Authors:** Manickam Sivakumar, Krishnan Venkatakrishnan, Bo Tan

**Affiliations:** 1 Department of Aerospace Engineering, Ryerson University, 350 Victoria Street, Toronto, ON M5B 2K3, Canada; 2 Department of Mechanical and Industrial Engineering, Ryerson University, 350 Victoria Street, Toronto, ON M5B 2K3, Canada; 3 On leave from Department of Sciences, Amrita School of Engineering, Amrita Vishwa Vidyapeetham, Ettimadai, Coimbatore 641105, India

## Abstract

In this study, MHz pulse repetition rate femtosecond laser-irradiated gold-coated silicon surfaces under ambient condition were characterized by scanning electron microscopy (SEM), transmission electron microscopy (TEM), X-ray diffraction analysis (XRD), and X-ray photoelectron spectroscopy (XPS). The radiation fluence used was 0.5 J/cm^2 ^at a pulse repetition rate of 25 MHz with 1 ms interaction time. SEM analysis of the irradiated surfaces showed self-assembled intermingled weblike nanofibrous structure in and around the laser-irradiated spots. Further TEM investigation on this nanostructure revealed that the nanofibrous structure is formed due to aggregation of Au-Si/Si nanoparticles. The XRD peaks at 32.2°, 39.7°, and 62.5° were identified as (200), (211), and (321) reflections, respectively, corresponding to gold silicide. In addition, the observed chemical shift of Au 4*f *and Si 2*p *lines in XPS spectrum of the irradiated surface illustrated the presence of gold silicide at the irradiated surface. The generation of Si/Au-Si alloy fibrous nanoparticles aggregate is explained by the nucleation and subsequent condensation of vapor in the plasma plume during irradiation and expulsion of molten material due to high plasma pressure.

## Introduction

Nanostructures of Au, Au-Si alloy, and Si have been employed in micro and nanoelectromechanical systems [1], biosensors [2], and photonics [3,4]. The field-emission property [5] of Au-Si alloy structures is used for the fabrication of panels and displays. Au-Si alloy nanoparticles are increasingly relevant as they are used as catalysts in the growth of nanowires [6]. Recently, femtosecond lasers have proven to be a powerful tool for nanostructuring of bulk metals [7-10]. Femtosecond laser pulses have also been used for precise nanostructuring of thin films with minimal thermal side effects [11-13]. The ultrafast excitation of materials controls the deposited energy in the material with femtosecond pulses. As a result, nanostructures with spatial resolution smaller than the wavelength of radiation can be generated. Although interaction of laser radiation with gold, gold-silicon thin films that lead to the formation of microbumps/nanojet structures have been studied [11,14,15], investigations on the Au-Si/Si fibrous nanoparticles aggregate formation using femtosecond laser radiation under ambient condition have not been reported. In the previous studies, synthesis of self-assembled weblike fibrous nanoparticles aggregate structures, nanofibers, and nanoscale tips with bulk semiconductor, metallic, and dielectric materials using femtosecond laser radiation under ambient condition was reported [8,16-18]. The fibrous structure generation is explained by nucleation and condensation of plasma plume generated during the irradiation process. It was comprehended that generation of nanofibrous structures can significantly be controlled by laser radiation fluence, laser interaction time, and pulse repetition rate [16]. This study is aimed to investigate the synthesis of Si/Au-Si alloy nanoparticles aggregate with femtosecond laser irradiation of Au-coated silicon samples and the influence of laser interaction time on composition of structures. The plausible mechanism underlying Au-Si alloy nanoparticles aggregate generation will also be discussed. The irradiated sample surfaces are characterized using scanning electron microscopy (SEM), transmission electron microscopy (TEM), and X-ray diffraction analysis (XRD). The chemical composition of nanoparticles aggregate is further analyzed using X-ray photoelectron spectroscopy (XPS).

## Experimental methods

The laser source used (λ = 1030 nm) is a direct-diode pumped Yb-doped fiber oscillator/amplifier system capable of delivering a maximum of 15 W average power with a pulse repetition rate ranging from 200 kHz to 25 MHz. The beam profile is Gaussian and the spot diameter is (10 μm) measured at 1/*e*^2^. The samples used were gold-coated (thickness 200 to 400 nm) silicon wafers. The laser beam is focused on the sample surface with a lens of focal length of 71 mm and scanned using a computer-controlled galvanometer to produce arrays of spots. The experiments were carried out in air at atmospheric pressure. The radiation fluence used was 0.5 J/cm^2 ^at a pulse repetition rate of 25 MHz with an interaction time of 1 ms and with the pulse width of 214 fs. Three sets of samples were prepared with same experimental conditions. One set was used for SEM analysis. The other set was used for XRD analysis followed by TEM investigations. Third set was used for XPS analysis. XRD measurements were performed with a Cu Kα radiation (λ = 0.154184 nm). The diffractograms were recorded using Bruker detector from 20° to 70°. To transfer the nanostructures to TEM grids, the samples were sonicated in isopropanol solution. A drop of the nanoparticles aggregate dispersed solution was placed on the copper grid and allowed to dry in air. XPS measurements were carried out on a Thermo Scientific K-Alpha XPS spectrometer. A monochromatic Al Kα X-ray source was used, with a spot area (on a 90° sample) of 400 μm.

## Results and discussion

**Figure 1:**
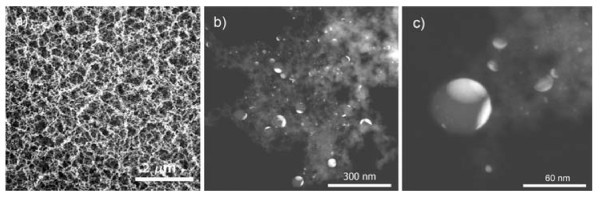
**SEM and TEM micrographs of the sample surface irradiated at laser radiation fluence (0.5 J/cm^2^) with interaction time (1 ms) and the nanoparticles aggregate respectively**. **(a) **surface featuring weblike nanoparticles around the laser spot, **(b, c) **Si/Au-Si nanoparticles in the aggregate structure.

**Figure 2:**
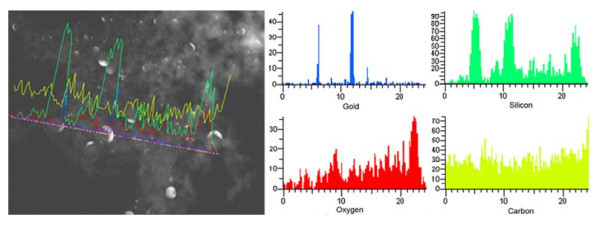
**TEM-EDX analysis of the nanoparticles in the aggregate structure**.

SEM micrographs of the sample surfaces around the laser-irradiated spots are shown in Figure 1a. Weblike fibrous nanoparticles aggregate with certain degree of porosity is observed in and around the laser-irradiated spots with all processing parameters used. SEM/EDX analysis of the nanostructure shows the presence of gold, silicon, and oxygen. In addition to Au-Si, nanoparticles of amorphous silicon which are comparatively smaller are also observed (Figure 1b). The size of Au-Si nanoparticles in the fibrous nanoparticles aggregate structure is bigger than that of Si particles as evidenced from TEM analysis (Figure 1b, c). Moreover, the different nanoparticles are intermixed with each other in the aggregate. TEM/EDX analysis of the nanoparticles aggregate revealed the composition Au-Si nanoparticles. The relative amount of silicon and gold in Au-Si nanoparticle varies with different particles (Figure 2).

**Figure 3:**
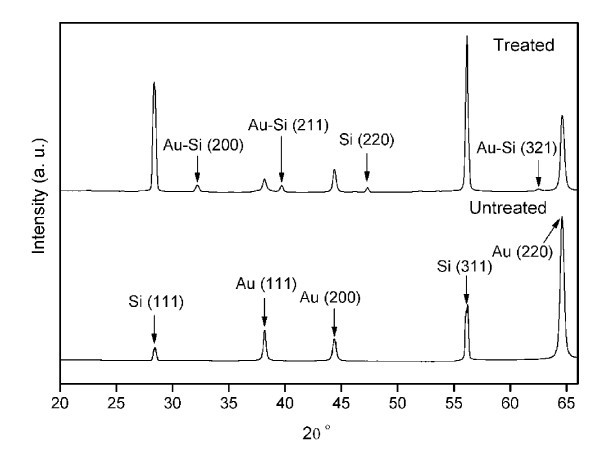
**X-ray diffractograms of both treated and untreated samples**. The peak at 39.7° in the treated sample is attributed to the Au-Si alloy phase.

X-ray diffractograms of both treated and untreated samples were performed with Cu Kα radiation (λ = 0.1541848 nm) [19] (Figure 3). The peaks 32.2°, 39.7°, and 62.5° were identified as (200), (211), and (321) reflections, respectively, corresponding to JCPD file for Au81Si19 (JCPD 39-0735). Further peaks at 38.2°, 44.38°, and 64.6° were identified to originate from (111), (200), and (220) planes of Au (JCPD 04-0784), respectively. The average Au-Si particles size is calculated from the full-width at half-maximum (FWHM) of the diffraction peaks using the Debye-Scherrer formula [20]*D *= *k*λ/βcosθ, where *D *is the mean grain size, *k *is a geometric factor (= 0.89), λ is the X-ray wavelength, β is the FWHM of diffraction peak, and θ is the diffraction angle. The grain sizes of Au-Si calculated from the peaks 32.2° and 39.7° are 26 and 55 nm, respectively.

**Figure 4:**
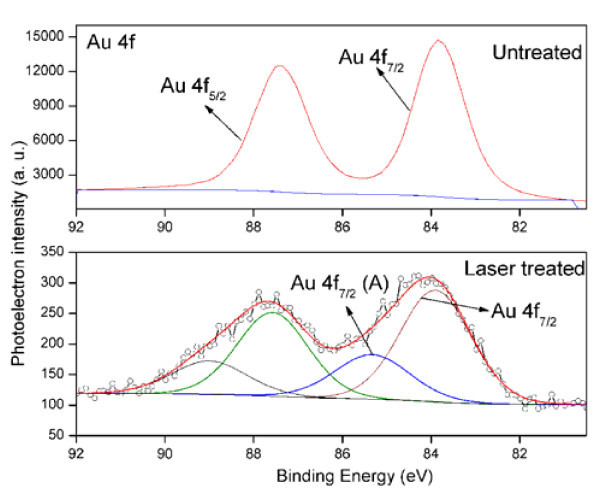
**XPS spectra of untreated and laser treated samples for Au 4*f *system**.

**Figure 5:**
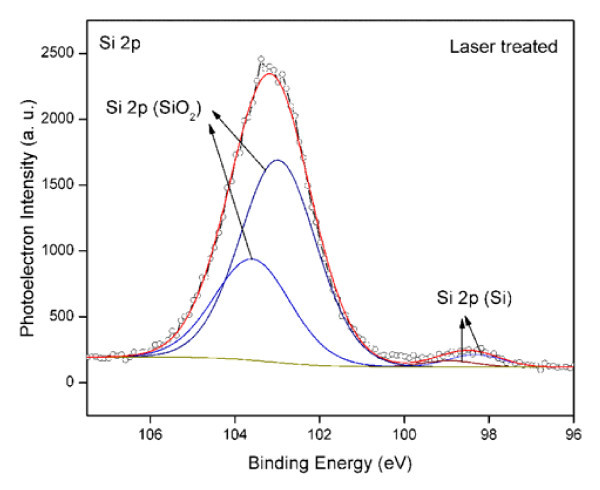
**XPS spectra of laser treated samples for Si 2*p *system**.

The chemical state of Au, Si, and oxygen atoms for both untreated and laser-treated samples was investigated by XPS. The correction of the XPS spectra for the charge accumulation was performed using C 1s peak (BE = 284.6 eV), which can be ascribed to contaminant hydrocarbons. Figure 4 shows XPS spectra of Au 4*f *line obtained for the untreated and laser-treated samples. For the untreated samples, the peaks of Au 4*f*_7/2 _and Au 4*f*_5/2 _lines are located at BE = 83.82 and 87.4 eV, which correspond to elemental gold. Laser-irradiated samples showed broadened peaks of Au 4*f*_7/2 _and Au 4*f*_5/2 _lines. Deconvolution of these lines showed the presence of two peaks in each line. For instance, the peaks of Au 4*f*_7/2 _line appear at 85.33 eV, which is a characteristic of metallic gold, and at 83.9 eV, 1.43 eV higher in BE can be ascribed to silicide [21-23]. Moreover, the 83.9 eV peak is due to the interaction of gold with silicon at the interface [24] by laser irradiation, resulting the formation of gold silicide in the nanoparticles aggregate. The presence of elemental gold with laser-irradiated samples is due to untreated areas of the sample around the laser spot above which nanoparticles aggregate was formed. Since the thickness of gold layer deposited on sample surface is about 200 to 400 nm, XPS analysis of untreated sample has not showed the silicon peak. However, investigations on the laser-irradiated sample surface revealed the presence of Si 2*p *line. The deconvoluted spectrum of the laser-treated sample is shown in Figure 5. The peaks are due to silicon nanoparticles in the aggregate structure.

Irradiation of metal films using femtosecond laser radiation results in fast nonequilibrium processes such as laser melting and film disintegration [7,25]. The energy from laser radiation is absorbed by the conduction band electrons and results in a sharp increase in electronic temperature near the irradiated front surface. Since the heat capacity of electrons in a metal is much smaller than that of lattice, an ultrashort laser pulse can heat electrons to a very high temperature while leaving the lattice relatively cool. The fast temperature-dependent electron heat conduction leads to the redistribution of the deposited energy within the film. This process occurs simultaneously with a more gradual energy transfer from electrons to the lattice vibrations due to electron-phonon coupling. The time duration of energy transfer and the ensuing equilibrium processes depends on electron-phonon coupling in gold. For Au, the coupling is 2.1 × 10^16 ^W/m^3 ^K, and the energy is transferred to the lattice within 15 ps. The equilibrium between hot electrons and lattice takes place with a time limit of up to 50 ps [25]. During irradiation, first few pulses alter the gold film and significantly increasing the absorption of this modified surface for the subsequent pulses [9]. At 25 MHz pulse repetition rate, the pulse separation time is 40 ns, Au film reaches high temperature due to accumulation of heat from successive pulses. Laser pulse repetition rate plays a significant role in generating these structures due to cumulative heating. At MHz laser pulse repetition rate, the delay between successive pulses is comparable to the critical time of nucleation [8]. Besides, repetition rate helps to sustain the molten liquid thereby maintaining the plasma and nanoparticles agglomeration. Taking into consideration the different plume components, formation of gold nanoparticles with various sizes under femtosecond laser irradiation is explained by nucleation and condensation of vapor in the plasma plume and explosion of molten material due to high plasma pressure [17].

The melting point of Au is 1063°C while for Si it is 1414°C. Although for an alloy which contains 81% Au and 19% Si, the melting point is 359°C, called the eutectic point. The formation of Au-Si alloy catalyst is explained via a Vapor-Liquid-Solid (VLS) process [6] during the synthesis of Si nanowires. In this experiment, since the laser radiation fluence (0.5 J/cm^2^) used is much above the ablation threshold of Au (0.2 J/cm^2^) with multiple pulses [26], gold may diffuse into the silicon substrate to form an alloy at the interface [27]. This alloy layer at the interface started melting due to cumulative heating by the subsequent laser pulses. The expulsion of molten alloy material results in the formation of alloy nanoparticles. Once the alloy layer is depleted further irradiation ablates the underlying silicon substrate and generates the plasma. At this point, the silicon nanoparticles are generated by nucleation and condensation of vapor in the plasma [8] and agglomerates with Au-Si alloy nanoparticles to form weblike nanofibrous structure. Although irradiation of molten alloy nanoparticles by subsequent laser pulses may increase their temperature, it is not supporting the growth of silicon nanowires [27]. The relative proportion of alloy nanoparticles in the aggregate nanostructure is mainly determined by the laser interaction time. This is implicitly understood from the SEM/EDX analysis, which shows the atomic percent of gold and silicon as a function of interaction time in the fibrous aggregate structure. In contrast to normal VLS process [6], where the source of semiconductor is supplied as vapor phase, in this case both gold and silicon are originated from the gold-coated solid silicon substrate. Upon laser irradiation, molten Au-Si alloy layer is formed and transforms into molten nanoparticles and agglomerates as solid weblike self-assembled fibrous structure. In other words, the fibrous nanoparticles aggregate process can be regarded as Solid-Liquid-Solid [27] process. In this experiment, Si/Au-Si fibrous nanoparticles aggregate is generated in a single step under ambient condition. The existence of Au-Si alloy in the nanoparticles aggregate is corroborated from XRD, TEM/EDX, and XPS analysis. Moreover, the size of alloy nanoparticles calculated from the peaks of XRD diffractogram matches well with the size observed from TEM micrographs.

## Conclusions

A simple method of generating Si/Au-Si alloy nanoparticles aggregate using MHz pulse repetition rate femtosecond laser radiation under ambient conditions is reported. The generation of nanoparticles aggregate is explained by nucleation and condensation of vapor in the plasma plume and expulsion of molten material due to high plasma pressure. This technique could be extended to generate other metal-semiconductor alloy nanostructures. Further studies are required to find the influence of film thickness and laser processing parameters on nanostructure generation.

## Abbreviations

SEM: scanning electron microscopy; TEM: transmission electron microscopy; VLS: vapor-liquid-solid; XPS: X-ray photoelectron spectroscopy; XRD: X-ray diffraction analysis.

## Competing interests

The authors declare that they have no competing interests.

## Authors' contributions

SM carried out laser processing of the samples, characterisation and drafted the manuscript. KV conceived of the study, and participated in its design and co ordination. BT conceived of the study, and participated in its design and co ordination. All authors read and approved the final manuscript.
